# Lack of specificity associated with using molecular beacons in loop mediated amplification assays

**DOI:** 10.1186/s12896-019-0549-z

**Published:** 2019-08-01

**Authors:** Patrick Hardinge, James A. H. Murray

**Affiliations:** Cardiff School of Biosciences, Cardiff, Museum Avenue, Cardiff, CF10 3AX UK

**Keywords:** Loop-mediated amplification (LAMP), Molecular beacons, DNA, Genetically modified (GM) crops, Fluorescence

## Abstract

**Background:**

Loop mediated isothermal amplification of nucleic acid templates is a rapid, sensitive and specific method suitable for molecular diagnostics. However the complexity of primer design and the number of primers involved can lead to false positives from non-specific primer interactions. Standard methods of LAMP detection utilise the increasing concentrations of DNA or inorganic pyrophosphate and therefore lack specificity for identifying the desired LAMP amplification. Molecular beacons used in PCR reactions are target specific and may enhance specificity with LAMP.

**Results:**

We present a potential molecular beacon approach to LAMP detection targeting the single stranded region between loops, and test this for LAMP molecular beacons targeting the 35S promoter and NOS terminator sequences commonly used in GM crops. From these studies we show that molecular beacons used in LAMP, despite providing a change in fluorescent intensity with amplification, appear not to anneal to specific target sequences and therefore target specificity is not a benefit of this method. However, molecular beacons demonstrate a change in fluorescence which is indicative of LAMP amplification products. We identify the LAMP loop structure as likely to be responsible for this change in signal.

**Conclusions:**

Molecular beacons can be used to detect LAMP amplification but do not provide sequence specificity. The method can be used to determine effectively LAMP amplification from other primer-driven events, but does not discriminate between different LAMP amplicons. It is therefore unsuitable for multiplex LAMP reactions due to non-specific detection of LAMP amplification.

**Electronic supplementary material:**

The online version of this article (10.1186/s12896-019-0549-z) contains supplementary material, which is available to authorized users.

## Background

Isothermal nucleic acid amplification methods represent an attractive alternative to PCR technologies which are reliant on thermocycling equipment. There are numerous isothermal methods [1, 2], but the most prevalent in the scientific literature is Loop-mediated amplification (LAMP) first described over a decade ago [3].

The LAMP reaction utilises 4 or more primers complementary to 6 or more target sequences. At the initiation of the amplification hairpin forming forward and backward inner primers (FIP and BIP) strand invade the double stranded DNA target. These primers anneal and are extended by a polymerase with strong displacement activity. The nascent strands are displaced by further primers (F3 and B3) enabling the subsequent formation of double hairpin structures from which amplification can cycle. Further primers can be added targeting the single stranded DNA hairpin loop structures [4] or the stem regions between loops [5] to accelerate the DNA amplification. LAMP amplification has been used for many pathogen detection strategies due to rapid reaction times, high sensitivity, robustness to contaminants and specificity.

The specificity is inherent from the use of multiple primers but this can also mean that non-specific primer interaction is an issue. The false positives that these interactions can produce are primarily a result of LAMP primer design and the reaction conditions, but also the non-specific nature of some of the detection methods employed. Methods for detection of LAMP amplification have mainly focused on determining the increasing concentrations of DNA or inorganic pyrophosphate for both real time and end point assays and will inherently detect LAMP and potentially also non-specific DNA synthesis. Such methods include SYBR Green [6], BART bioluminescent reporter [7], turbidimetry [8] and specific dyes [9].

Recent work investigating the use of molecular beacons with LAMP [10] suggested that the problem of non-specific amplification could be solved for real-time assays through their use. An endpoint low melt temperature molecular beacon with LAMP assay has also been shown [11]. Molecular beacons for use in PCR were first described in 1996 [12] in which the beacon has complementary arms 5 nucleotide long and a 15 nucleotide long probe loop. A fluorophore attached to the 5’ end is quenched due to fluorescence resonance energy transfer (FRET) by the proximity of a quencher at the 3’ end. In the presence of specific target DNA the probe anneals and undergoes conformational change and fluorescence. No change is observed in the absence of specific target DNA and the fluorophore would remain quenched.

The thermodynamics and kinetics of molecular beacons for PCR have been studied [13, 14] and recommendations for arm length, probe length, melt temperature and GC content for different applications [15] have been provided. In thermocycling, the PCR primers and molecular beacon bind at the annealing temperature to the target, and before the end of this stage the fluorescence is generated and acquired. Increasing the temperature to the extension temperature liberates the molecular beacon allowing PCR cycling to continue. The denaturing stage renders the DNA single stranded to continue the process, and the increase in fluorescence with each cycle is observed in the presence of increasing amounts of specific template DNA.

In LAMP amplification this process is different due to two factors; namely the single temperature throughout the assay and the displacement polymerase. The recent study [10] with MB-LAMP showed that fluorescence increased in real time together with LAMP template amplification. This was achieved with molecular beacon lengths of 25-45 base pairs, concentrations of 0.6-1.0 picomoles/microlitre and reaction temperatures of 60-65 degrees C.

The mechanism by which increased specificity is conferred from the annealing of the LAMP molecular beacon to the specific target sequence is however unclear. Since assays involve a constant temperature and use a displacement polymerase that would likely displace the molecular beacon. We show here that molecular beacons indeed detect LAMP amplification but this does not require specific annealing to the specific target sequence. However the increase in fluorescence does appear to be linked to increasing concentration of LAMP amplicons and therefore the specificity is greater than methods that report on DNA amplification only since a positive signal is indicative of successful LAMP amplification. The problem of posssible non-specific amplification remains due to the failure of the LAMP molecular beacons to anneal specifically to the target sequence. Nevertheless, the low fluorescence output from the LAMP molecular beacon method enables the quantification of a wider dynamic range of target concentrations to be assayed in comparison to detection with an intercalating fluorescent dye.

The detection and quantification of genetically modified materials in food is essential to comply with legislation and two of the most common markers of GMO contamination are the cauliflower mosaic virus 35S promoter and nopaline synthase terminator from *Agrobacterium tumefaciens*, used to express the transgenes. Here we target these transgenic elements in maize event Bt11 in a mass fraction with wild type maize. We have designed molecular beacons for the 35S promoter and NOS terminator sequences to complement LAMP primers previously established for amplification [16–18].

Our work initially focused on probing the forward loop structure of LAMP amplicons, but this precluded the use of accelerating loop primers [4]. We therefore concentrated on the stem region between loops to enable the forward loop primer to be included for faster amplification. This work revealed that whilst molecular beacons detect LAMP amplification products, they do so irrespective of amplified sequence. They can therefore be used to detect LAMP amplicon, but not the nature of the amplified sequence.

## Methods

### DNA templates

**Fig. 1:**
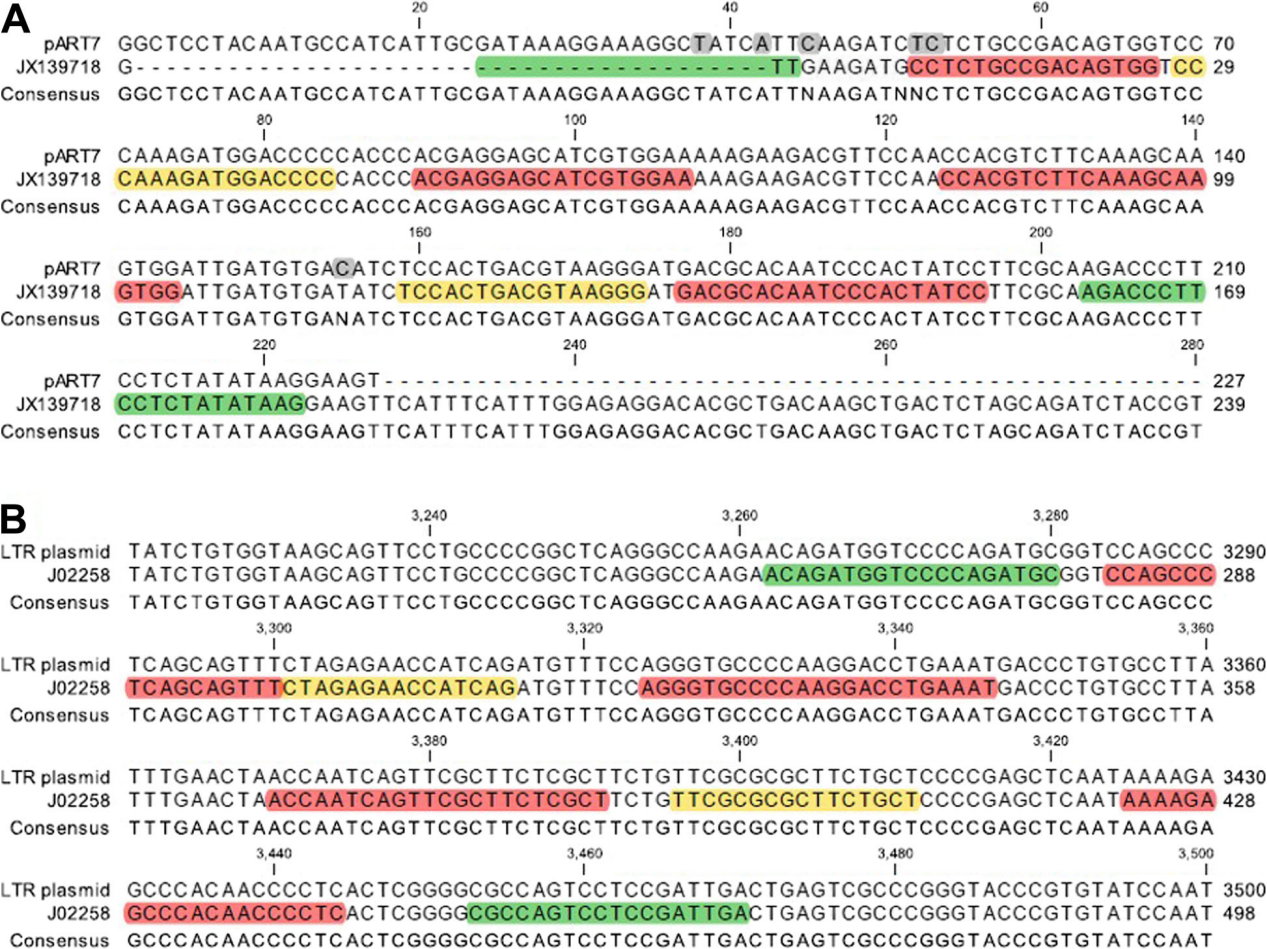
Position of LAMP primers for 35Sp **a** and LTR **b** with plasmid template DNA sequences. Displacement primers denoted F3 and B3 are labelled in green with the loop primers LoopF and LoopB labelled yellow. The two components of the LAMP primers FIP and BIP are labelled red. The grey highlights for the pART7 sequence show the mismatches of this variant of the 35S promoter sequence

The plasmid pART7 [19] containing a cauliflower mosaic virus 35S promoter (35Sp) sequence variant was linearised and initially quantified by NanoDrop spectrophotometry and Agilent Bioanalyzer for a defined target concentration. A further plasmid containing a retroviral LTR sequence from Moloney Murine Leukaemia Virus (accession number: J02258.1) was targetted by LTR LAMP primers. The sequences of the plasmids for the LAMP primers are displayed in Fig. 1. Transgenic GM maize event Bt11 certified reference material (CRM) from the Institute for Reference Materials (Geel, Belgium) was purchased from Fluka GMBH (Buchs, Switzerland) with a defined mass fraction of 5.0 percent w/w with wild type maize. Bt11 contains the 35S promoter sequence (35Sp) from the cauliflower mosaic virus and the nopaline synthase terminator (NOSt) from *Agrobacterium tumefaciens*. The genomic DNA was extracted using the Promega Wizard genomic DNA purification kit (Madison, United States) and quantified using a Qubit fluorimeter (Life Technologies, UK).

Two further DNA templates were synthesised by IDT (Coralville, Iowa, USA) firstly to create a simplified target for the 35S promoter LAMP primers using a sequence length and structure appropriate to the FIP, BIP, LoopF and LoopB primers only. The second oligonucleotide was designed to match the length of the first primer but without loop forming sequences. The sequences are shown in Table 1.

**Table 1:** Sequence information for artificial 35Sp LAMP templates

Target, Notation, Version	Template Sequence (5’ to 3’)
35Sp BIP Dumbbell, LOOPS, PH	CACGTCTTCAAAGCAAGTGGGGATAGTGGGAT
	TGTGCGTCCCCTTACGTCAGTGGACCACTTGC
	TTTGAAGACGTGGTCTAGATTCCACGATGCTC
	CTCGGGGGTCCATCTTTGGGCCACTGTCGGCA
	GAGGCGAGGAGCATCGTGGAA
35Sp partial sequence, LINEAR, PH	TTGCGATAAAGGAAAGGCCATCGTTGAAGATG
	CCTCTGCCGACAGTGGTCCCAAAGATGGACCC
	CCACCCACGAGGAGCATCGTGGAAAAAGAAGA
	CGTTCCAACCACGTCTTCAAAGCAAGTGGATT
	GATGTGATATCTCCACTGACGT

35Sp templates synthesised and PAGE purified by IDT (Coralville, Iowa, USA). Both oligonucleotides are 150 bases in length, diluted to 10 micromolar and stored at minus 20 degrees C

### LAMP amplification

Loop-mediated amplification uses primers with different functions to enable isothermal DNA amplification. The initiation of the process requires the target strand invasion by the forward and backward inner primers (FIP and BIP) with synthesis of the nascent DNA from these primers utilising the displacement activity of Bst polymerase. The initiation event also requires the displacement primers F3 and B3 to displace the products of FIP and BIP extension which can in turn start the cycling between dumbbell LAMP structures (a dynamic structure with single stranded DNA loops at each end). Additional forward and backward loop primers, LoopF and LoopB, anneal and extend from the target loop structures to increase the synthesis of DNA and inorganic pyrophosphate for accelerated detection strategies. This role can also be met by the incorporation of STEM primers that bind between the F1 and B1 primer sequences. The position of the different sets of LAMP primers is shown for the two plasmid targets in Fig. 1.

### Primer design and synthesis

Oligonucleotide primers for LAMP nucleic acid amplification were synthesized and HPLC purified by Sigma Aldrich (Poole, UK). Primers were hydrated with molecular grade water to 100 micromolar and stored at minus 20 degrees C. LAMP primers for Moloney Murine Leukaemia Virus LTR were designed with the assistance of PrimerExplorer v4 from Eiken. The primer sequences are shown in Table 2.

**Table 2:** LAMP primers for 35S promoter, NOS terminator and LTR sequences

Target, Type, Notation, Version	Primer Sequence (5’ to 3’)
35Sp, Displacement, F3, GK	CTTATATAAGAGGAAGGGTCT
35Sp, Displacement, B3, GK	GATAAAGGAAAGGCTATCATT
35Sp, LAMP, FIP, GK	CCACGTCGGCAAAGCAAGTGG-TTTT-
	GGATAGTGGGATTGTGCGTC
35Sp, LAMP, BIP, DL	TTCCACGATGCTCCTCG-TTTT-CCTCTGCCGACAGTGG
35Sp, Loop, LoopF, DL	TCCACTGACGTAAGGG
35Sp, Loop, LoopB, DL	GGGGTCCATCTTTGGG
NOSt, Displacement, F3, DL	CGCGATAATTTATCCTAGTTTG
NOSt, Displacement, B3, DL	CGTTCAAACATTTGGCAAT
NOSt, LAMP, FIP, PH	GCATGACGTTATTTATGAGA-TTTT-
	TCGCGCTATATTTTGTTTTCTA
NOSt, LAMP, BIP, PH	CATGCTTAACGTAATTCAACA-TTTT-
	TGAATCCTGTTGCCGGTC
NOSt, Loop, LoopF, DL	GATTAGAGTCCCGCAATTATAC
NOSt, Loop, LoopB, DL	AAATTATATGATAATCATCGCAA
LTR, Displacement, F3, PH	ACAGATGGTCCCCAGATGC
LTR, Displacement, B3, PH	TCAATCGGAGGACTGGCG
LTR, LAMP, FIP, PH	ATTTCAGGTCCTTGGGGCACCCCAGCCCTCAG
	CAGTTTC
LTR, LAMP, BIP, PH	ACCAATCAGTTCGCTTCTCGCTGAGGGGTTGT
	GGGCTCTT
LTC, Loop, LoopF, PH	CTGATGGTTCTCTAGA
LTC, Loop, LoopB, PH	TTCGCGCGCTTCTGCT

Primers targeting the 35S promoter and NOS terminator have been previously described and are attributable to David Lee (DL) [16] and Guy Kiddle (GK) [17]. The Moloney Murine Leukaemia Virus LTR LAMP primers are described for the first time

### LAMP molecular beacons

The design of the 35Sp 5nt LoopF MB for LAMP followed in part the guidelines for molecular beacon design for PCR amplification; arm length 5 to 7 nucleotides, probe sequence 15 to 30 nucleotides, Tm above the annealing temperature of the assay, 5’ fluorophore not attached to a guanine nucleotide with a 3’ non-fluorescent quencher. The length of the probe sequence was restricted by the single stranded elements of the LAMP loop or STEM. The 35Sp LoopF 5nt MB is similar in structure to the first described molecular beacon by Tyagi and Kramer in 1996 [12], with 5nt arms and 15nt probe sequence. The primer sequences are shown in Table 3 with associated melt temperatures (Tm), GC content (percentage) and nucleotide length. The LAMP molecular beacons were synthesized and HPLC purified by Sigma Aldrich (Poole, UK).

**Table 3:** LAMP molecular beacons designed for 35S promoter and NOS terminator sequences

Target, Type, Vers., Tm, GC, length	Primer Sequence (5’ to 3’)
*ompW*, LBP, WL, 69.8, 69.2, 25	[6FAM]AGCGGCTGCAGCCCTACTAGCCGCT[Dabcyl]
35Sp LoopF, 5nt FAM, 76.8, 66.6, 24	[6FAM]CCGAGCCACTGACGTAAGGCTCGG[OQA]
35Sp stem, 4nt FAM, 63.1, 61.1, 18	[6FAM]CCACTGGAACGTCTGTGG[BHQ1]
35Sp stem, 5nt FAM, 72.7, 65.0, 20	[6FAM]CGCACTGGAACGTCTGTGCG[BHQ1]
35Sp stem, 5nt JOE, 72.7, 65.0, 20	[JOE]CGCACTGGAACGTCTGTGCG[BHQ1]
35Sp stem, 6nt FAM, 77.9, 68.1, 22	[6FAM]CGGCACTGGAACGTCTGTGCCG[BHQ1]
35Sp stem, 7nt FAM, 82.0, 70.8, 24	[6FAM]CCGGCACTGGAACGTCTGTGCCGG[BHQ1]
NOSt stem, 5nt FAM, 66.0, 43.4, 23	[6FAM]CGCACTTACATGTTAATTGTGCG[BHQ1]
NOSt stem, 5nt FAM, 66.0, 43.4, 23	[JOE]CGCACTTACATGTTAATTGTGCG[BHQ1]

The fluorophores used were 6-carboxyfluoroscein (6FAM) and 4’, 5’-dichloro-2’, 7’-dimethoxy-5(6)-carboxyfluorescein (JOE). The quenchers used were 4-(dimethylaminoazo)benzene-4-carboxylic acid (Dabcyl), Onyx Quencher A (OQA) and Black Hole Quencher 1 (BHQ1). Molecular beacon version WL was designed by Liu et al. 2017 [10] to detect part of the *ompW* gene from *Vibrio cholerae*

### Fluorescent LAMP amplification

DNA samples were amplified and detected in real time on a Qiagen (Hilden, Germany) Rotor-Gene thermal cycler acquiring to the green channel. Unless otherwise stated, all reagents are supplied by Sigma Aldrich (Poole, UK). The reaction chemistry for LAMP and amplification detection was 1X isothermal buffer (NEB, Massachusetts, United States), 300 micromolar each dNTP, 0.8 micromolar Betaine, 0.5 micromolar SYTO9 Green, 0.32 units per microlitre Bst polymerase v2.0 warm start (NEB), 0.8 micromolar each LAMP primer, 0.4 micromolar each Loop primer, 0.2 micromolar each displacement primer and molecular grade water for a reaction volume of 20 microlitre. The parameters were set for 60 cycles of 60 seconds at 60 degrees C unless otherwise stated. Melt temperature analysis provided data between 60 and 95 degrees C. LAMP amplification detection with SYTO9 was substituted with LAMP molecular beacons and supplemented with molecular grade water if required.

### PCR amplification

DNA samples were amplified using PCR on a Qiagen Rotor-Gene thermal cycler with detection of SYTO9 on the green channel and detection of the 35Sp STEM 5nt JOE MB on the yellow channel. The primers shown in Table 4 where used to flank the JOE labelled MB without overlapping target sequences. The reaction chemistry was 1X Qiagen Taq PCR mastermix, 0.5 micromolar SYTO9 Green, 1.0 micromolar 35Sp 5nt JOE MB and 2.0 micromolar each PCR primer with molecular grade water for a reaction volume of 10 microlitre. The thermocycling used the following conditions; initial denaturation for 3 min at 94 degrees C, followed by 40 cycles of 30 seconds at 94 degrees C, 30 seconds at 55 degrees C and 60 seconds at 72 degrees C. Melt curve analysis acquired stepwise between 30 and 95 degrees C. Fluorescence acquisition for the JOE labelled MB was on the yellow channel at the end of the annealing step. SYTO9 detection used the green channel with acquisition at the end of the extension step.

**Table 4:** PCR primers for 35S promoter amplification. The M35F primer is derived from Wu et al. 2014 [20] and the M3R is from Fernandez et al. 2005 [21]. Both primers were synthesised by Sigma with HPLC purification

Target, Type, Notation	Primer Sequence (5’ to 3’)
35Sp, Forward, M25F	CATCATTGCGATAAAGGAAAGGC
35Sp, Reverse, M3R	TCTTGCGAAGGATAGTGGGATT

### Data analysis

Sequences were aligned using CLC sequence viewer 7 and graphically displayed in pdf format. The Basic Local Alignment Search Tool (BLAST) from NCBI was used for sequence specificity and LAMP primers and LAMP molecular beacons were designed with the assistance of Eiken Primer Explorer v4 and Primer3 [22] respectively. Rotor-Gene thermocycling results were analysed using Rotor-Gene 6000 software v1.7, converted with TeeChart Office and graphically displayed using GraphPad Prism 7. Statistical analysis used Microsoft Excel to calculate the average Ct and standardard deviation results and GraphPad Prism 7 for the coefficient of determination (R^2^) values.

## Results

### Specificity of LAMP molecular beacon targeting the forward loop sequence

**Fig. 2:**
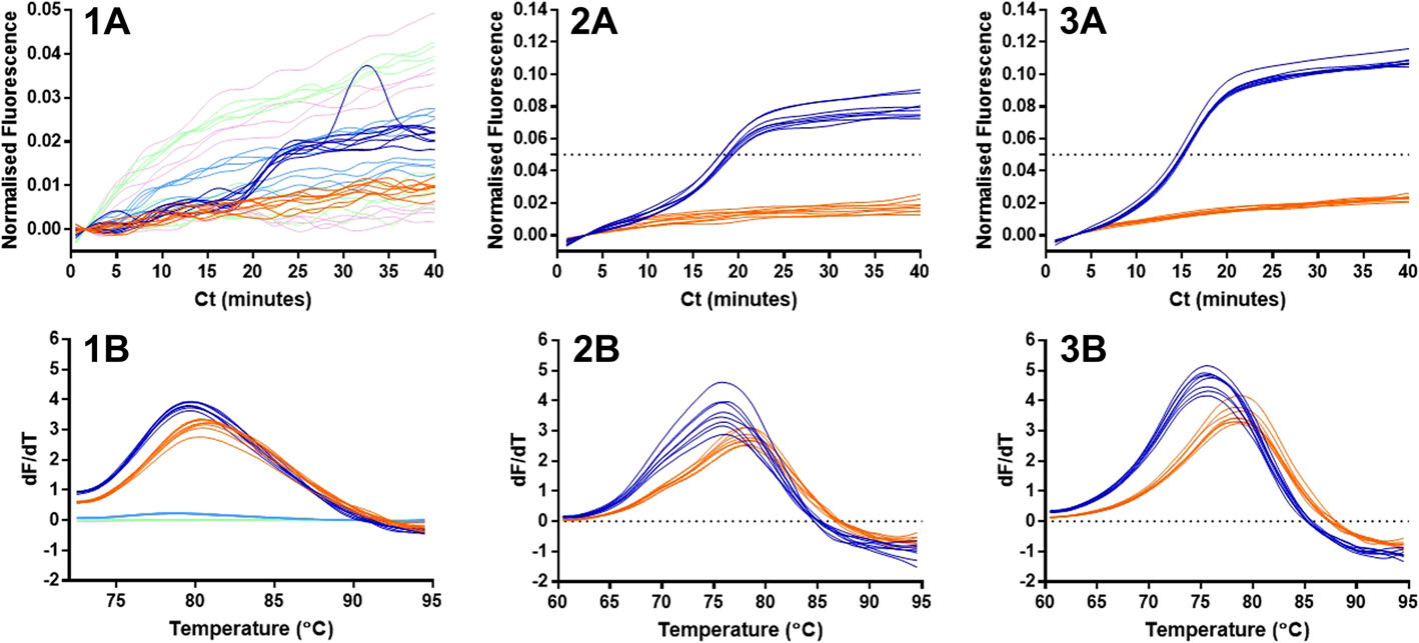
LAMP amplification with 35Sp LoopF molecular beacon. LAMP primers designed to amplify part of the 35S promoter sequence detected by fluorescence from a LAMP molecular beacon targeting the LoopF sequence. 1A shows the fluorescence for dilutions of the 35Sp LAMP MB; 125 nanomolar dark blue, 12.5 nanomolar light blue, 1.25 nanomolar light green, 125 picomolar pink and NTC in orange. 2A denotes the fluorescent output against cycles of 1 min at 63 degrees C for 35Sp LAMP amplification excluding the LoopF oligonucleotide (positive samples with template in blue and NTC in orange). 3A charts the fluorescent output against cycles of 1 min at 63 degrees C for 35Sp LAMP amplification with the inclusion of the LoopF oligonucleotide. LAMP molecular beacon used at a concentration of 0.32 micromolar for these two assays. Melt curve analysis shown in 1B to 3B with peaks inverted

**Fig. 3:**
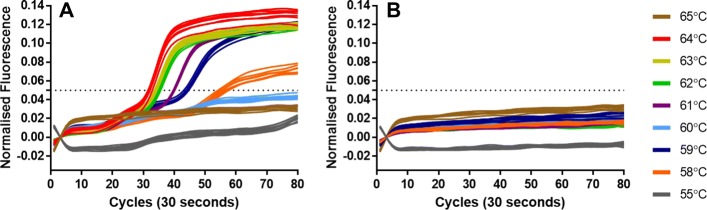
Amplification curves of 35Sp 5nt STEM molecular beacon at different temperatures. LAMP assay temperature incrementally from 55 degrees C to 65 degrees C for positive template and NTC, (**a**) shows the increase in fluorescence of the FAM fluorophore attached to the 35Sp 5nt STEM molecular beacon at various temperatures and (**b**) shows the corresponding NTCs for each assay. The positive and negative samples at various temperatures for the 35Sp 4nt STEM MB, 35Sp 6nt STEM MB and for 35Sp 7nt STEM MB are shown in Additional file 1: Figure S1

We designed a LAMP molecular beacon targeted to the forward loop sequence of a variant of the 35S promoter from Cauliflower mosaic virus (Fig. 1), with a probe sequence of 14 nucleotides and complementary arms of 5 nucleotides. Using this, we noted an increase in fluorescence with a 125 nanomolar concentration of the MB for the positive sample in relation to the negative sample (Fig. 2; 1A). The use of this primer in a 63 degrees C 35Sp LAMP assay at a concentration of 320 nanomolar with the omission of the LoopF primer from the reaction mix resulted in a clear difference between the positive and negative samples (Fig. 2: 2A). A further experiment with the inclusion of the LoopF primer, which targets the same sequence as the LAMP molecular beacon, produced a clearer separation between the positive and negative samples. Moreover the average Ct values improved by almost 4 min, presumably due to the increased amplification from the LoopF primer despite possible interference from the MB binding the same sequence. The dynamic environment of LAMP amplification which involves strand displacement by Bst polymerase, makes it unlikely that the molecular beacon remains annealed to the Loop sequence, given the positive effect of the LoopF primer on LAMP kinetics. Melt curve analysis revealed that the positive samples have a lower melt temperature than the negative samples; the difference is about 2 degrees C for the assay without the LoopF primer (Fig. 2; 2B) and as an average 3 degrees C for the assay with the LoopF primer (Fig. 2; 3B).

### LAMP molecular beacons targeting the STEM region and optimal reaction temperature

To allow for rapid amplification from the unencumbered use of Loop primers, we therefore designed LAMP molecular beacons to target the region of the LAMP amplicon called the STEM region, lying between the hairpin loops. The probe length of these molecular beacons remained fixed at 10 nucleotides but the length of the self-complementary arms was increased from 4 to 7 nucleotides incrementally. Positive and negative samples were amplified using LAMP reactions containing each of these molecular beacons over a temperature range of 55 to 65 degrees C. All negative template controls showed negative results below the 0.05 normalised fluorescence threshold level set for the assays. The negative results followed a similar line with the exception of assays at 55 degrees C in which the fluorescence from the molecular beacons was reduced (Fig. 3).

The LAMP molecular beacon results from the positive samples showed stronger signals from the 35Sp 5nt STEM MB (Fig. 3; A) and 35Sp 6nt STEM MB (Additional file 1: Figure S1; 2A) than for the 35Sp 4nt STEM MB (Additional file 1: Figure S1; 1A) and 35Sp 7nt STEM MB (Figure S1;3A). The optimum temperature giving the fastest and most differentiated result compared to the negative samples was 64 degrees C. The dynamic equilibrium between quenched fluorescence and unquenched fluorescence due to the interaction between the arms of the molecular beacons is expected to cause increased baseline fluorescence at higher temperature. This appears to be less evident as the length of the arms increases therefore increasing the stability of the quenched form. The results for the positive samples indicate the molecular beacon with 5 nucleotide arms gives the strongest fluorescent signal relative to background and was selected for further optimisation.

### Limitations of molecular beacons with LAMP

**Fig. 4:**
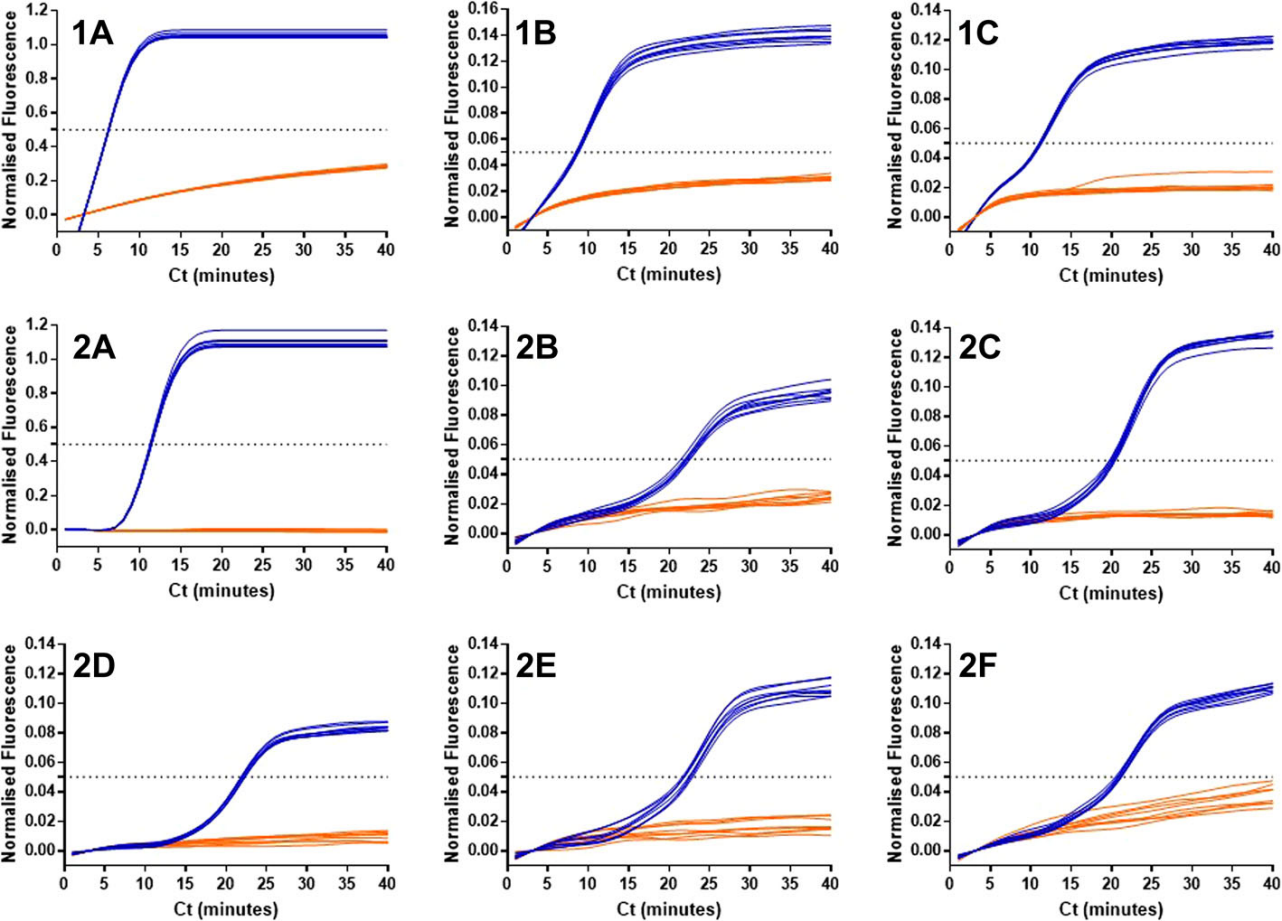
Lack of enhanced specificity with LAMP molecular beacons. 1A denotes 35S promoter LAMP amplification of an optimised 35Sp template with amplification detection using SYTO9 dye (positive template control in blue and NTC in orange), 1B shows detection of 35Sp LAMP amplification with 35Sp 5nt MB and 1C shows detection of 35Sp LAMP amplification with NOSt 5t MB. 2A denotes LTR LAMP amplification of a plasmid using SYTO9 dye, 2B shows detection of the LTR LAMP amplification with 35Sp LoopF MB, 2C shows LTR LAMP with NOSt 5nt STEM molecular beacon detection, 2D shows LTR LAMP with 35Sp 4nt STEM MB, 2E with 35Sp 5nt STEM MB and 2F with 35Sp 6nt STEM MB

It is assumed that the LAMP molecular beacons add increased specificity to the LAMP assay by exhibiting fluorescence when associated with a particular amplified target sequence. In a series of experiments displayed in Fig. 4, this assumption was investigated.

35S promoter LAMP primers were used to amplify a standard concentration of template under identical conditions with the exception of SYTO9 (Fig. 4; 1A), 35Sp 5nt STEM MB (Fig. 10; 1B) and NOSt 5nt STEM MB (Fig. 4; 1C) incorporated for amplification detection. As expected the LAMP amplification was detected by the SYTO9 dye and by the LAMP molecular beacon designed for the 35Sp LAMP STEM sequence. However, unexpectedly the 35Sp LAMP amplification was also detected by the LAMP molecular beacon designed to a NOS terminator LAMP STEM sequence not present in the target. The probe sequence of this primer is 5’-TTACATGTTAATT-3’ compared to 5’-TGGAACGTCT-3’ for the probe sequence of the 35Sp LAMP STEM molecular beacons. Further investigation into this lack of specificity used LAMP amplification of a retroviral LTR sequence to provide an alternative LAMP template for the various LAMP MBs. Detection of the LTR LAMP amplification was detected by SYTO9 (Fig. 10; 2A), by the 35Sp 5nt LoopF MB (Fig. 4; 2B), by the NOSt 5nt STEM MB (Fig. 4; 2C) and by 35Sp STEM molecular beacons 4nt, 5nt and 6nt (Fig. 4); 2D to 2F respectively). We conclude the detection of LAMP amplicons by this type of molecular beacons is not sequence dependent.

### Specificity of LAMP molecular beacon targeting the backward loop sequence

**Fig. 5:**
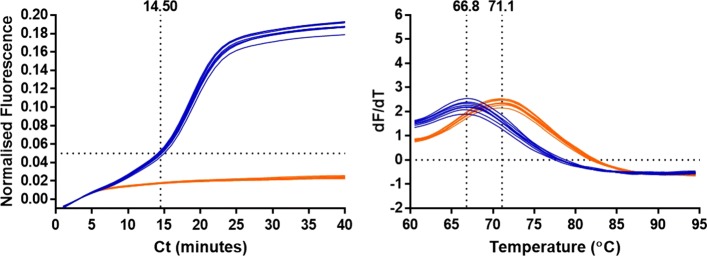
Lack of enhanced specificity with LBP LAMP molecular beacon. LAMP amplification of 35Sp template in blue and non-template control in orange with detection of amplification with a FAM fluorophore labelled molecular beacon designed for the ompW gene of *Vibrio chlorerae* [10]. Fluorescent output against cycles of 60 seconds at 60 degrees C and melt curve analysis post amplification

Liu et al. 2017 [10] described LAMP molecular beacons targeting the forward loop sequence of the *ompW* gene from *Vibrio chlorerae*. Their optimised molecular beacon had a 13 nucleotide probe sequence with two complementary arms of 6 nucleotides terminated with a 5’ FAM fluorophore and a 3’ DABCYLl quencher. The optimal temperature was 63 degrees C at a concentration of 320 nanomolar. We used this molecular beacon with the 35Sp LAMP amplification of a 35Sp artificial template to investigate the specificity of this method (Fig. 5).

The high concentration of the 35Sp artificial template was rapidly amplified using 35Sp LAMP primers with detection of fluorescence from the LBP LAMP molecular beacon. The fluorescence from the non template control remained sub-threshold and there was clear separation between the positive and negative samples after less than 10 min. The melt curve fluorescence showed a lower temperature for the molecular beacon with LAMP amplification of 66.8 degrees C compared to the molecular beacon without LAMP amplification of 71.1 degrees C.

### LAMP molecular beacons with looped or linear DNA templates

**Fig. 6:**
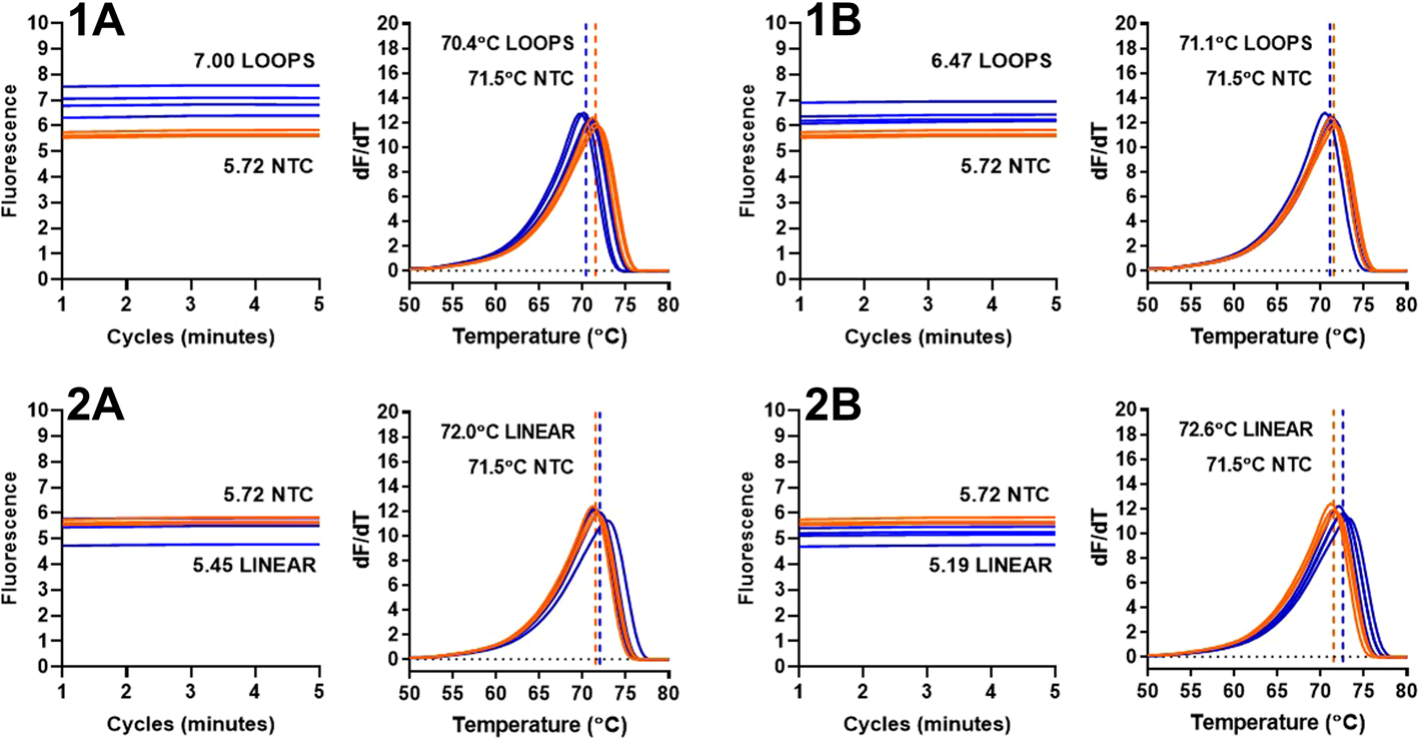
Fluorescent signal from MB-LAMP with looped or linear DNA.35Sp oligonucleotide designed for ‘dumbbell’ looped structure undiluted (1A) and ten-fold dilution (1B) in blue, NTC in orange. Fluorescence from LBP molecular beacon in real-time at 60 degrees C for 5 min and melt curve analysis. Average fluorescence and melt temperature derived from replicates. 35Sp oligonucleotide of the same length without loops undiluted (2A) and ten-fold diluted (2B) in blue with NTC in orange. Fluorescence acquisition from LBP molecular beacon at 60 degrees C for 5 min and melt curve analysis

Two oligonucleotide templates and ten-fold dilutions were combined separately with 0.8M betaine, 1x isothermal buffer (NEB) and the LBP LAMP molecular beacon. The fluorescence from the FAM fluorophore was acquired after 60 seconds for a total of 5 min at 60 degrees C. The oligonucleotide designed to form loops, based on one of the 35Sp dumbbell structures, showed an increase in fluorescence when compared to the NTCs with undiluted and diluted template (Fig. 6 1A, 1B). The melt curve analysis for these assays showed a reduction in melt temperature of template compared to NTC. The increase in real-time fluorescence and decrease in melt temperature were reduced by template dilution.

The other oligonucleotide template designed as a partial sequence of the 35S promoter without loop forming sequences showed a decrease in fluorescence for both the undiluted and diluted template compared to the NTCs during real-time acquisition, and an increase in the melt temperature comparison (Fig. 6 2A, 2B). The increased fluorescence from the LAMP molecular beacon in the presence of the ‘dumbbell’ template and the reduced melt temperature match the results seen in Fig. 5 with increasing concentrations of loop DNA structures during LAMP amplification. This effect was not observed with the linear DNA template.

### Optimal concentration for the 35Sp 5nt STEM molecular beacon at 64 degrees C

**Fig. 7:**
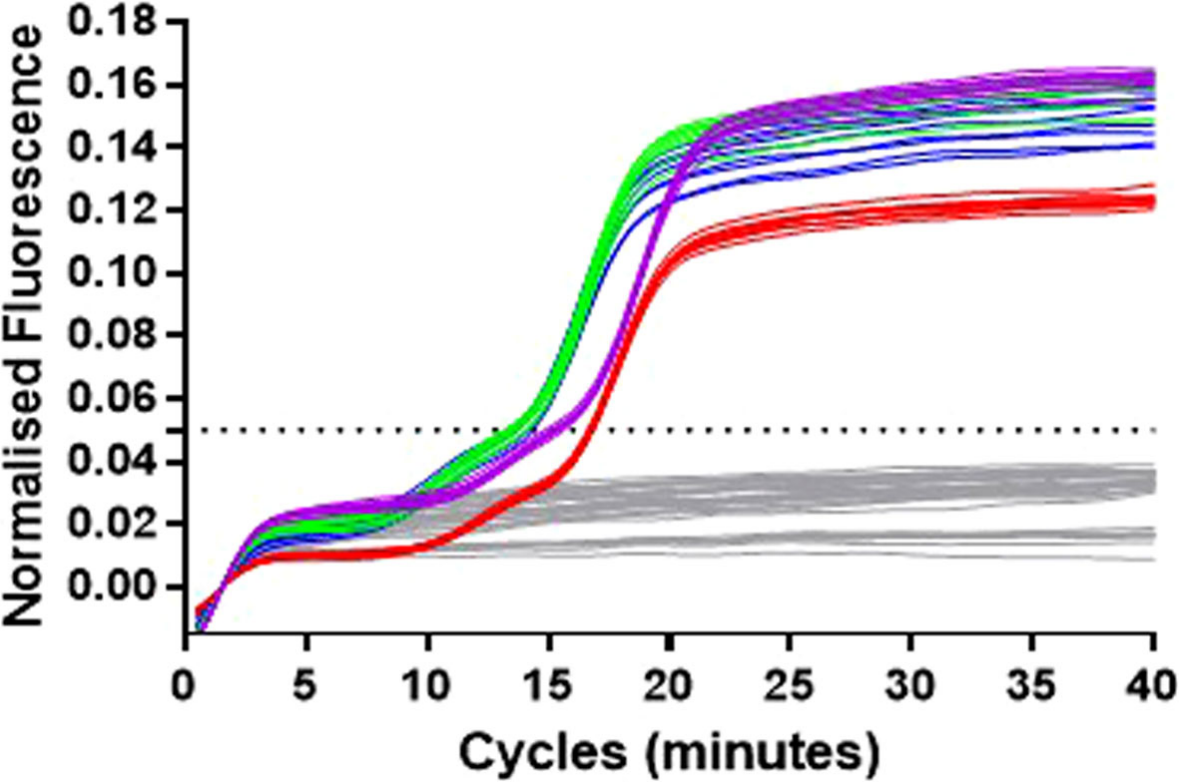
Optimal concentration of the 35Sp 5nt STEM molecular beacon. Standard LAMP assay at 64 degrees C with linearised plasmid template and negative template samples for each molecular beacon concentration. Molecular beacon concentrations: 4.0 micromolar purple, 2.0 micromolar red, 1.0 micromolar green, 0.5 micromolar blue and NTC in grey

Standard LAMP reactions at 64 degrees C using controlled positive and negative samples were used to optimise the concentration of the 35Sp 5nt STEM MB at 0.5, 1.0, 2.0 or 4.0 micromolar. The results indicated faster results for the 0.5 and 1.0 micromolar concentrations (Fig. 7) with no signal from the negative template controls.

**Fig. 8:**
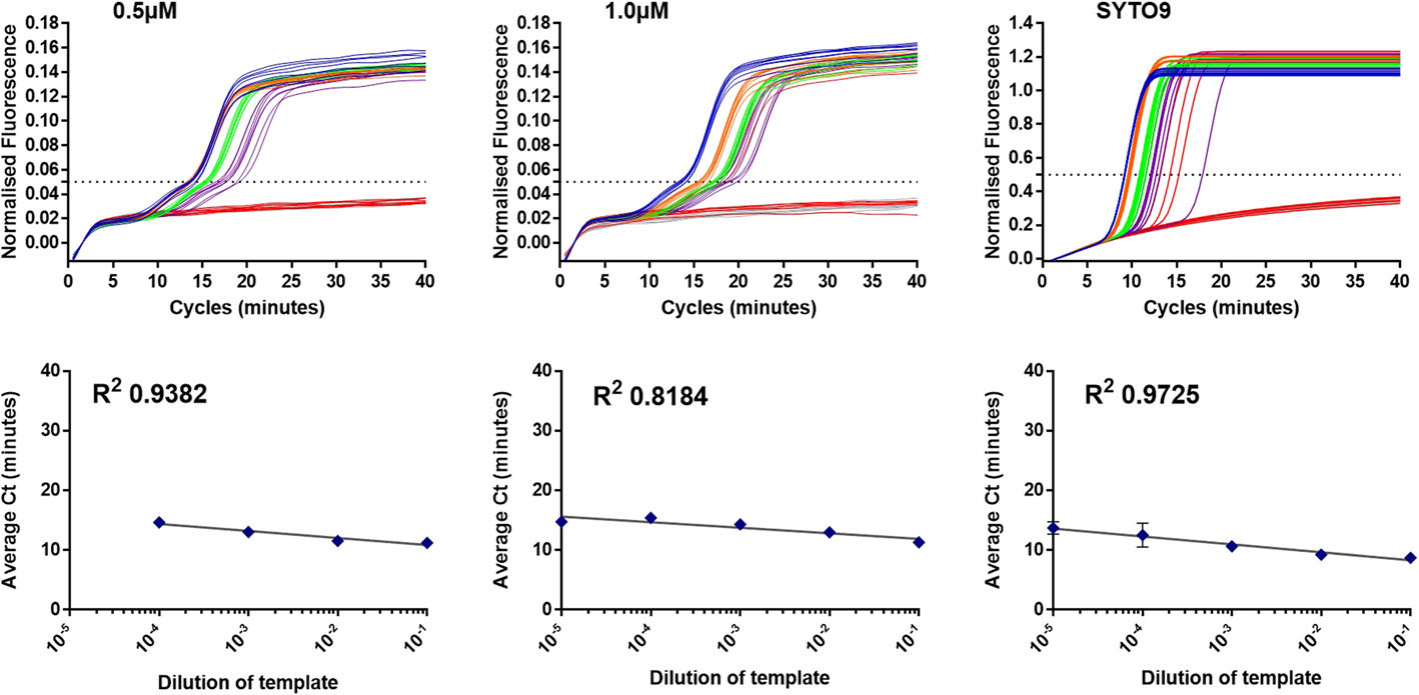
Variation and separation between replicates in quantification. Fluorescent output from 35Sp 5nt STEM MB or SYTO9 with 35Sp LAMP amplification of a serial dilution of plasmid template. Template dilutions: 10^-1^ dark blue, 10^-2^ orange, 10^-3^ light green, 10^-4^ purple, 10^-5^ red and NTC in grey. For each concentration of the molecular beacon the Ct value is derived from a set threshold of 0.05 (for the SYTO9 assay this value is 0.5). See Additional file 1: Figure S2 for 2.0 and 4.0 micromolar MB assays

To further investigate the optimum concentration for this LAMP molecular beacon dilution range, a serial dilution of the 35Sp plasmid template was prepared from 10^-1^ to 10^-5^ from stock concentration of 10^6^ copies/microlitre. All four LAMP molecular beacon concentrations previously tested (0.5 micromolar and 1.0 micromolar are shown in Fig. 8 and 2.0 micromolar and 4.0 micromolar are shown in the Additional file 1: Figure S2) were used for 35Sp LAMP assays and compared to identical assays with the molecular beacon replaced with the intercalating dye SYTO9 to report amplification. The results showed a reduced amplification frequency for the most dilute sample using the lowest concentration of 35Sp 5nt STEM molecular beacon and this concentration also had poor separation between the results for the higher concentrations of template.

Low variation between replicates was observed for the 10^-1^, 10^-2^ and 10^-3^ dilutions with LAMP amplification detected by SYTO9 fluorescence and all the dilutions of the LAMP molecular beacon. The increase in signal above baseline was observable for SYTO9 assays and MB assays 0.5 and 1.0 micromolar MB, but was delayed with higher MB concentrations of 2.0 and 4.0 micromolar, for all target DNA concentrations.

### Quantification using 35Sp 5nt MB

**Fig. 9:**
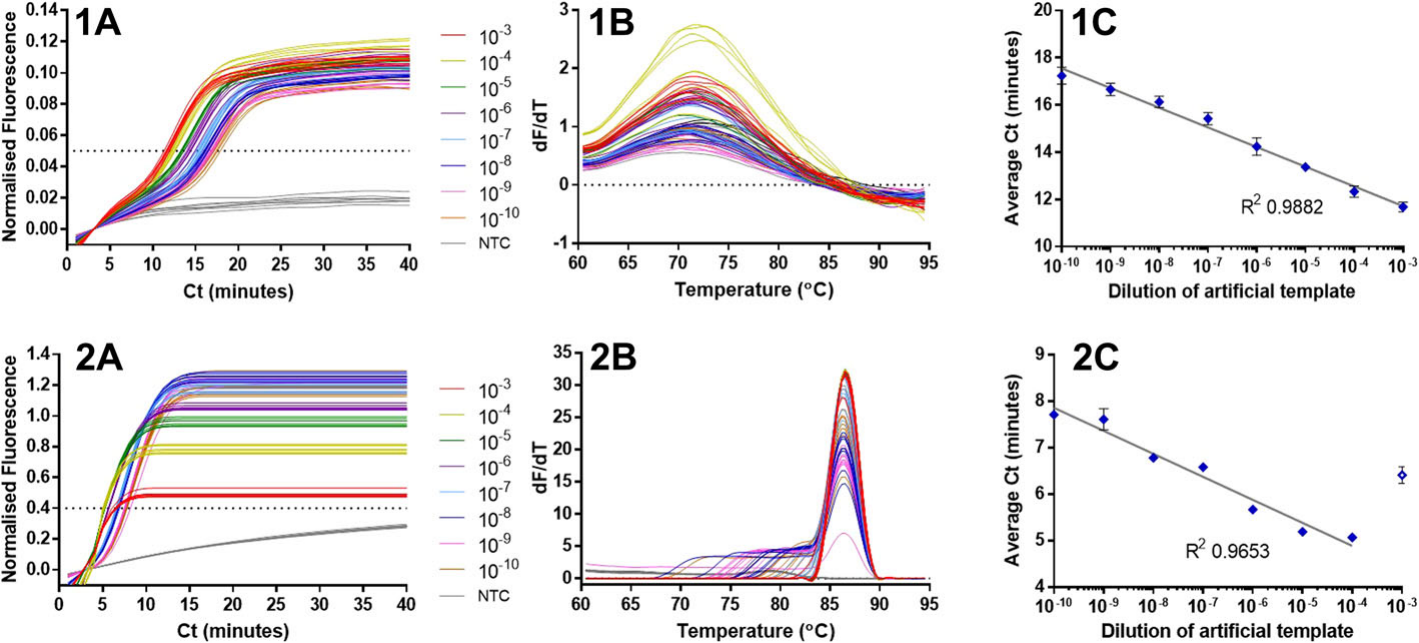
LAMP quantification with molecular beacon and SYTO9. The 35S promoter STEM 5nt molecular beacon was used with a serial dilution of an optimised artificial template in (1) and the same template dilutions were used with SYTO9 detection in (2). Normalised fluorescence for the dilutions series and NTC (A), Melt curve analysis for the dilution series and NTC (B) and average Ct against template dilution for the dilution series (C). The threshold was set at 0.05 for the LAMP MB and 0.4 for the SYTO9 detection. The peaks in 1B for the LAMP molecular beacon melt curve were inverted

The 35S promoter artificial LAMP template was serially diluted and the range 10^-3^ to 10^-10^ of the 10^12^ copies/microlitre stock, was investigated for the potential of the 35Sp LAMP molecular beacon method for quantification in comparison to the SYTO9 detection method. The results are shown in Fig. 9.

**Fig. 10:**
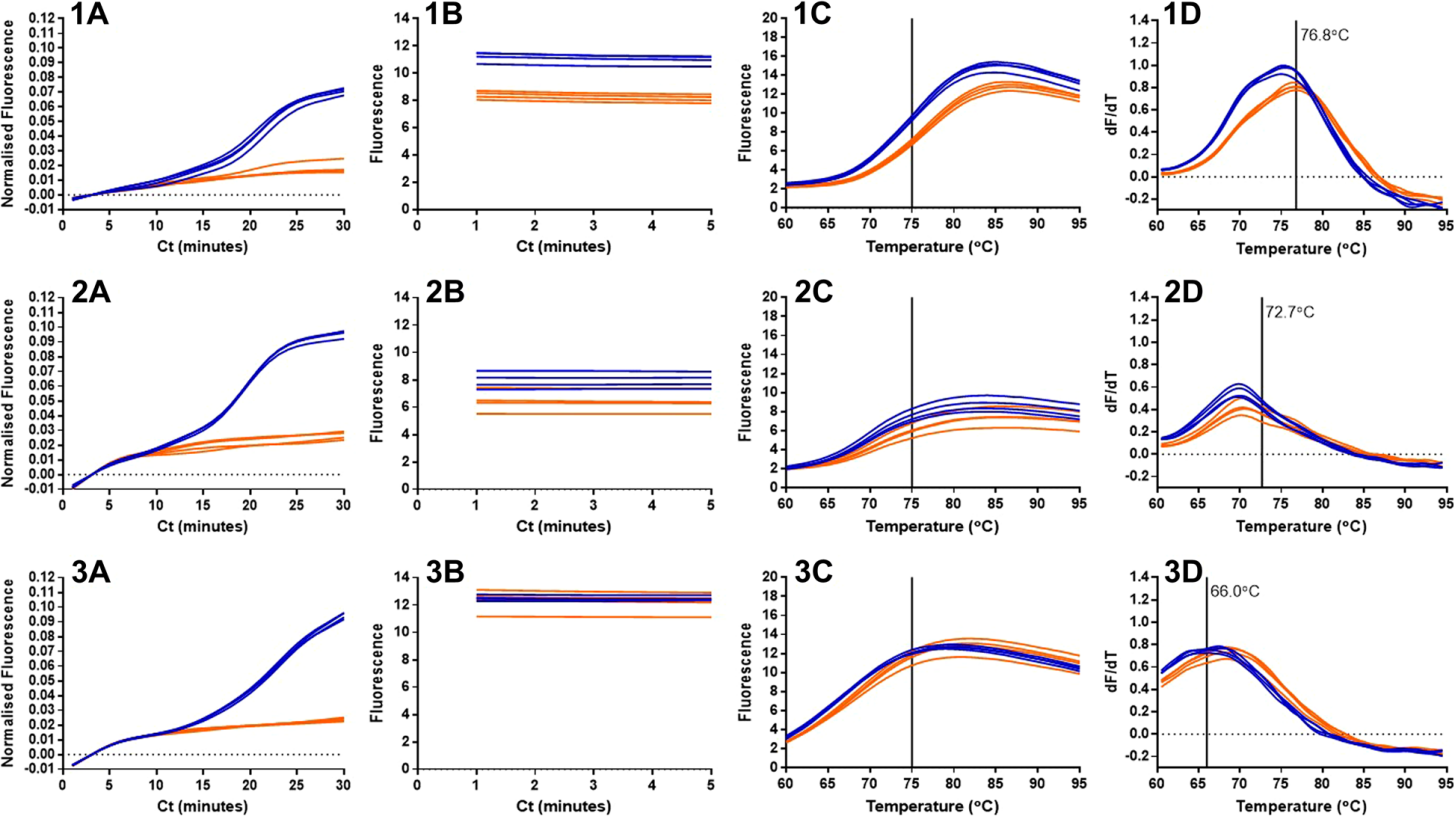
End point LAMP molecular beacons at 75 degrees C. LAMP amplification at 64 degrees C for 30 cycles of 60 seconds, followed by 95 degrees C for 5 min and 75 degrees C for 5 min with fluorescence acquisition. Melt curve analysis between 60 degrees C and 95 degrees C. Template in blue, NTCs in orange. (1) 35Sp LAMP amplification with 35Sp LOOP MB, (2) 35Sp LAMP amplification with 35Sp STEM MB and (3) NOSt amplification with NOSt STEM MB. (A) real-time normalised fluorescence during amplification, (B) end point fluorescence at 75 degrees C, (C) melt profiles showing separation at 75 degrees C and (D) melt curve analysis with the melt temperature (Tm) of the molecular beacon

The LAMP molecular beacon results show clear NTCs throughout the course of the assay and positive results distinguishable from the fluorescence baseline (Fig. 9; 1A). For each dilution there appears to be low variation between replicates and dilutions are clearly separate by their timing of signal increase. This is supported in the graph of average Ct against template dilution graph (Fig. 9; 1C) with the standard deviation between replicates shown as error bars and correlation of the data points to the semi-logarithmic trend line approaching an R^2^ value of 0.99. The melt curve analysis (Fig. 9; 1B) showed a range of average melt temperatures from 72.0 degrees C for the NTC and higher concentrations of template, down to 70.5 degrees C for the lowest concentration. LAMP amplification with detection using SYTO9 fluorescence of the same template dilution showed low variation between replicates and good separation between template dilutions. The wide ranging intensities of the fluorescence between the NTC and the most concentrated template dilution were problematic for the instrumentation with normalised fluorescence values ranging from approximately 0.5 for the 10^-3^ dilution to greater than 1.0 for the 10^-6^ to 10^-10^ dilutions. With the threshold set at 0.4 the average Ct values were plotted against the template dilutions (Fig. 6; 2C) and the highest concentration was excluded from further analysis due to the extremely high fluorescence producing an anomalous result. Lower variation was observed between replicates and lower average Ct values across the range than the LAMP molecular beacon results but the correlation of the data points to the semi-logarithmic trend line is lower (calculation excludes the data from the 10^-3^ dilution). We conclude that MBs can provide wide dynamic range quantification of LAMP amplification.

### Sensitivity

The quantified linear plasmid stock containing the 35S promoter was diluted to the equivalent of a single DNA copy per partition, and assayed by LAMP amplification using fluorescence detection with either 1 micromolar LAMP molecular beacon or SYTO9. The four LAMP molecular beacons designed to the STEM region were used to compare the sensitivity to the more established technique of intercalating fluorescent dye detection (Table 5).

**Table 5:** 35S promoter STEM molecular beacons and SYTO9 detection of low copy number assay

Detection Method	Amp. Freq.	Average Ct.	St. dev.	Fastest Ct.	Slowest Ct.
4nt STEM MB	25.80	31.06	12.74	21.91	83.33
5nt STEM MB	26.80	32.90	12.74	24.10	80.06
6nt STEM MB	32.40	35.26	10.65	25.02	65.16
7nt STEM MB	18.30	46.29	13.17	34.29	75.74
SYTO9	28.20	20.59	15.61	9.83	64.25

Diluted linear plasmid DNA containing the 35S promoter sequence assayed by 35Sp STEM molecular beacons of various lengths in comparison to the detection with SYTO9 fluorescence. LAMP assay at 64 degrees C with 1.0 micromolar LAMP molecular beacons and 72 replicates of 1 copy per partition. SYTO9 LAMP assay at 60 degrees C. The cycle parameters were set for 90 cycles of 60 seconds each cycle acquiring for FAM/SYTO9

The amplification frequency or percentage of positives from the number of replicates, was lowest for the 35Sp 7nt STEM MB at 18.3 percent, but the other LAMP MBs and the SYTO9 results were in a narrow range between 25.8 percent and 32.4 percent (Table 5). This is below the statistically predicted 63.2 percent amplification frequency for single copies per partition (0.3 to 0.4 copies per partition is indicated by these percentages). The results show that the LAMP MB and SYTO9 methods have the equivalent sensitivity to low copy number LAMP amplification. The average Ct and the fastest Ct both show that the 35Sp 7nt STEM MB produces the slowest results and SYTO9 is the faster method of detection. The standard deviation from the replicates for each assay were high within the range of 10.65 to 15.61; the highest value associated with the SYTO9 results. The slowest Ct values for each assay were all above 60 min highlighting the range of times that are observed with LAMP amplification at low copy number.

### LAMP molecular beacons end point assay

Molecular beacons have been used with LAMP to distinguish from false positives in an end-point assay (Liang Wan et al. 2017) [11]. We investigated whether the molecular beacons described here could be used for end-point LAMP amplification (Fig. 10).

The clearest separation between positive template and negative NTCs at 75 degrees C was for the 35Sp LAMP amplification with detection using the 35Sp LoopF MB (Fig. 10: 1B). Post amplification analysis of these samples showed that the fluorescence profile of the molecular beacon with template was different from that of the molecular beacon without template. Melt curve analysis showed that the MB without template had an average melt temperature of 76.7 degrees C close to the predicted Tm of 76.8 degrees C and yet the Tm for the MB with LAMP amplification of template was slightly reduced to 75.4 degrees C (Fig. 10: 1D). Lower average melt temperatures were also observed with the 35Sp STEM 5nt MB (predicted Tm of 72.7 degrees C) of 69.9 degrees C compared to 70.4 degrees C, and the NOSt STEM MB (predicted Tm of 66.0 degrees C) of 65.0 degrees C compared to 67.4 degrees C.

### PCR with LAMP molecular beacon; real time and end point

**Fig. 11:**
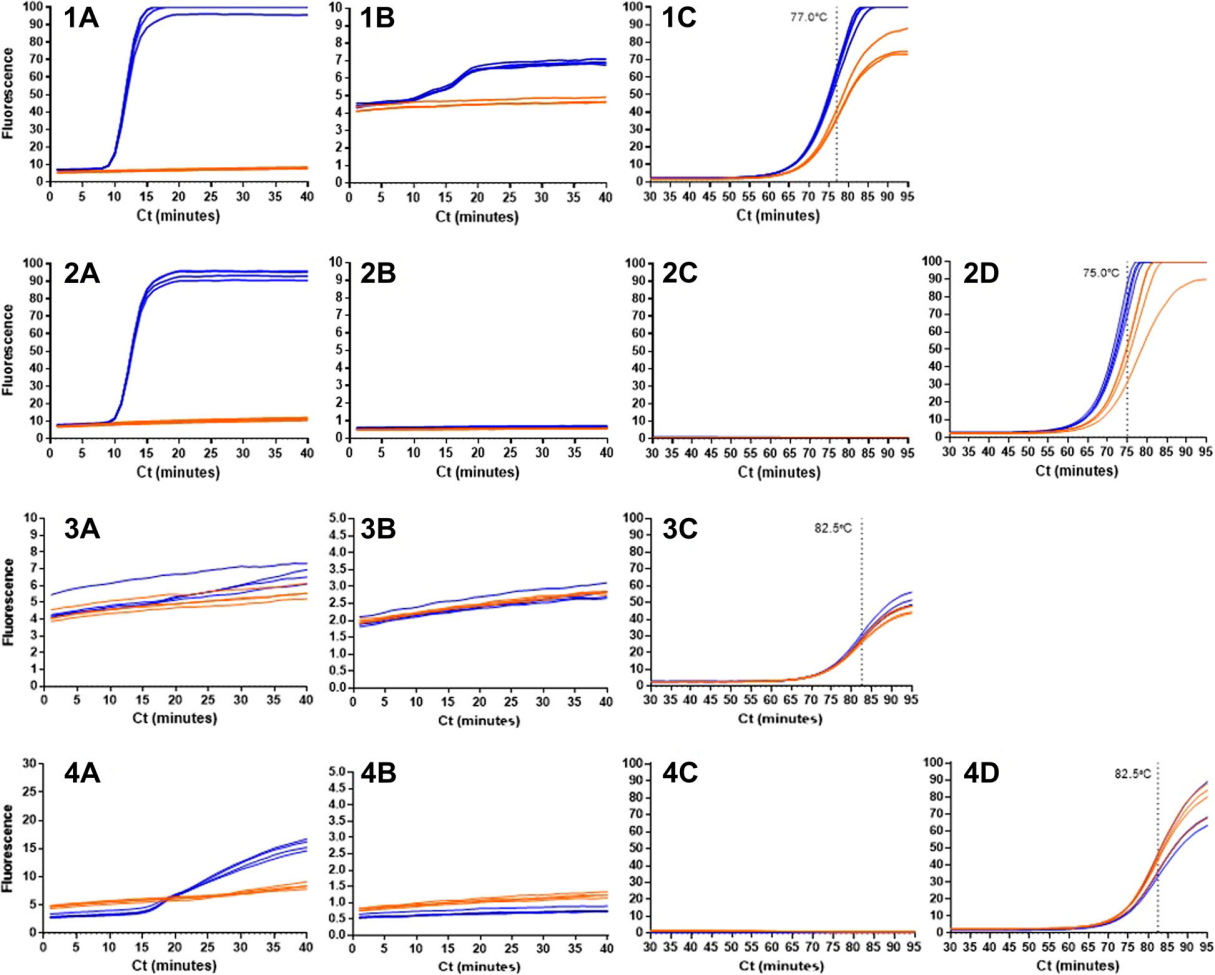
LAMP MBs with PCR and LAMP amplification 1 and 2 represent LAMP assays whereas 3 and 4 are PCR results. 1 and 3 represent assays with SYTO9 and the 35Sp 5nt MB, 2 and 4 include SYTO9 but no LAMP MB (template results in blue and NTCs in orange). Fluorescent detection of SYTO9 are shown in A. Detection of the presence or absence of the 35Sp 5nt JOE MB are shown in B with corresponding melt curves in C. 2D and 4D show the melt curve for the amplified samples from 2 and 4 after the post amplification addition of the LAMP MB

The typical structure of a PCR molecular beacon includes 5-7 nucleotide GC rich arms with a 22-30 nucleotide probe sequence [23] but the LAMP molecular beacons already described [10] and herein require shorter probe sequences of 10-14 nucleotides. PCR reactions were set up to include SYTO9 to report DNA amplification and a LAMP molecular beacon. Further PCR assays with SYTO9 but without a LAMP MB were run to produce an amplicon for end point detection through LAMP MB addition. The assays were repeated with LAMP amplification replacing PCR amplification (Fig. 11).

The results showed that the real time increase in fluorescence with the LAMP MB was not evident with PCR amplification. With LAMP amplification of the same sample (Fig. 11: 1 and 2) the SYTO9 detection results with LAMP MB (Fig. 11: 1A) and without LAMP MB (Fig. 11: 1B) showed similar fluorescent profiles indicating that the inclusion of the LAMP MB in the reaction mix did not significantly interfere with the LAMP amplification or detection. In contrast the inclusion of the LAMP MB appeared to interfere with PCR amplification; Fig. 11: 4A shows PCR amplification in the absence of the LAMP MB with a clear separation between positive and negative results, however this separation is not seen in Fig. 11: 3A with the inclusion of the LAMP MB. The detection of the JOE labelled MB was only evident with LAMP amplification in Fig. 11: 1B and not with PCR (Fig. 11: 3B). Melt curve detection of the LAMP MB was observed with both LAMP and PCR amplification in Fig. 11: 1C and 3C respectively, and with the addition of the LAMP MB post amplification in Fig. 11: 2D and 4D. We had noted in all LAMP experiments involving a MB the melt temperatures were lower in the presence of amplicon than for the MB alone in NTCs. This was unexpected since the MB has 10 nucleotides of homology to the target and self complementary arms of only 5 nucleotides. Moreover we did not observe this with PCR amplified samples (Fig. 11; 4D).

## Discussion

The use of a LAMP molecular beacon targeting the 35S promoter sequence and specifically the forward loop structure in the LAMP amplification was successful whether or not the LoopF primer was present, which would be competing for the sequence. Increased fluorescence and faster times were observed when the LoopF primer was included. The faster times can be explained by the accelerated LAMP reaction but the reporting of the faster time with increased fluorescence cannot be explained by the annealing of the probe and the primer to the same site due to the anticipated displacement activity of the polymerase. An alternative and potentially non-specific mechanism for the LAMP molecular beacon may be evident here.

We selected the single stranded DNA region between the loops in LAMP amplification to enable us to us to use the full set of primers and hence maximise the reaction kinetics and design MBs for 35S promoter and NOS terminator sequences commonly used in GM crops. Optimisation of these stem LAMP MBs resulted in a 64 degrees C reaction temperature, 1 micromolar probe concentration and 5 nucleotide arms (as predicted for PCR molecular beacons (Marras 2006 [15])). If it is the case that the LAMP molecular beacons do anneal to form a probe:target hybrid forcing the hybridised arms apart and increasing fluorescence, then the LAMP amplification would continue due to the displacement polymerase releasing the molecular beacon during extension. The dynamic process of LAMP amplification may explain why the fluorescence in the presence of template is low (but above that of the NTCs), because of the continuous annealing and displacement of the open molecular beacons which can then hybridise the arm sequences. A benefit of the low levels of fluorescence intensity for positive samples is the potentially high dynamic range of detection and quantification. Also the stem LAMP MB method achieved a similar high level of sensitivity in terms of amplification frequency for single copies per partition compared to SYTO9 detection.

**Fig. 12:**
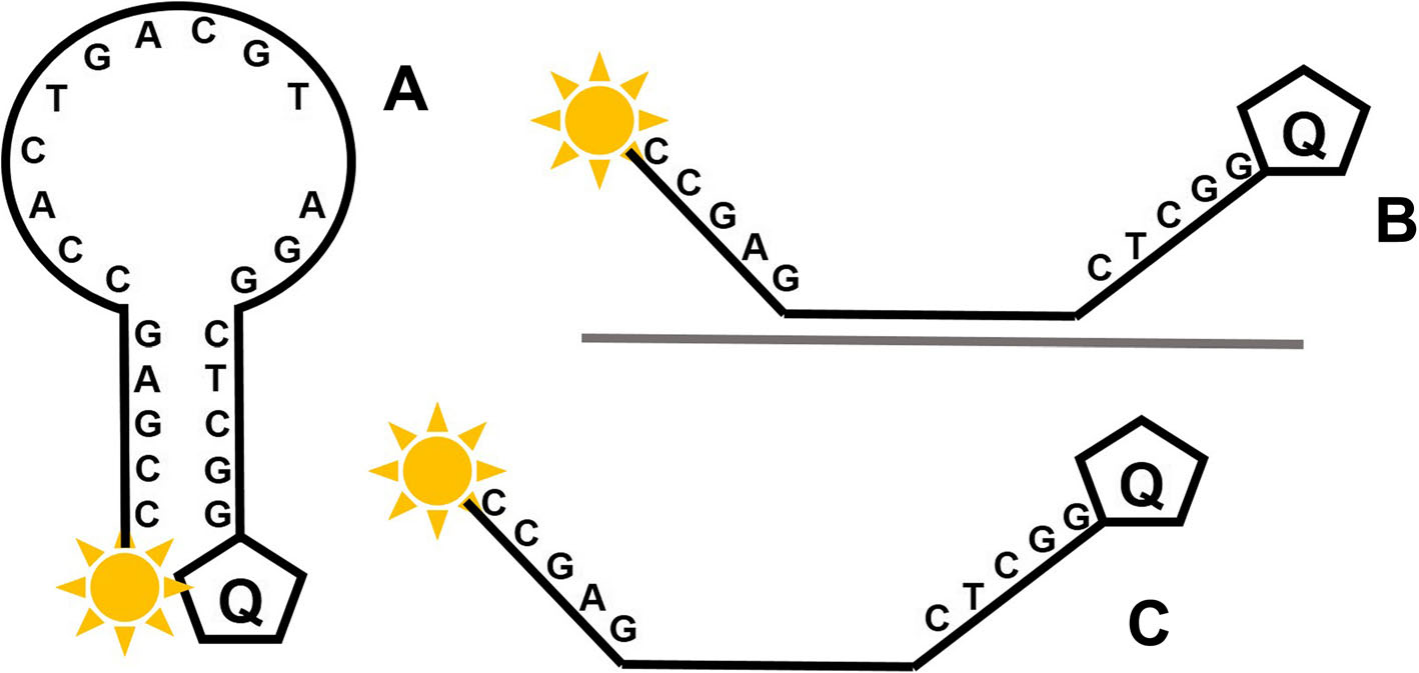
Molecular beacon structure and potential conformations. Molecular beacons are single stranded DNA oligonucleotides with a 3’ fluorophore and 5’ quencher. Hybridised arms ensure fluorescence is quenched in the absence of specific template to the probe sequence in the loop (**a**). In (**b**) the presence of a specific single stranded DNA sequence forms a more thermodynamically stable probe – template hybrid pulling the quencher away from the fluorophore and enabling fluorescence to be emitted. In (**c**) the molecular beacon is in ‘open’ formation which may be due to the assay temperature in equilibrium with the ‘closed’ form, or by interaction with LAMP amplification products

The increased specificity of LAMP molecular beacons derives from the assumption that a fluorescent signal can only be generated with the target amplification product. More precisely that the molecular beacon can only fluoresce after conformational change brought on by probe:target hybridisation. Non-specific amplification from the interaction between primers should therefore remain undetected. However our investigations showed that the detection of LAMP amplification without a specific molecular beacon binding can occur. This was first shown with 35Sp LAMP amplification detected by a 5 nucleotide arm molecular beacon with a 13 nucleotide probe sequence specific for the stem region of NOS terminator LAMP amplification. This probe sequence is not present in the 35S promoter amplicon. Further analysis on LAMP molecular beacons was conducted on the LAMP amplification of a retroviral LTR sequence of a plasmid DNA template. All the LAMP molecular beacons should have non-specificity to the amplified products however all the 35Sp and NOSt designed MBs produced positive results in the presence of LTR LAMP amplification and negative results with the NTCs. From this data the LAMP molecular beacons are not binding specifically to the LAMP amplicons and therefore the mechanism for the increase in fluorescence is directed towards the increase in DNA (Fig. 12). It is unclear whether this is related to an increase in total DNA or to the mechanism of LAMP amplification, but it does appear that the loop structures of LAMP amplification are important and our experiments with linear and looped DNA templates support this.

End point LAMP amplification detection with LAMP MBs was previously described (Liang Wan et al. 2017) [11] with low melt temperatures molecular beacons to mitigate a perceived interference with LAMP amplification. We found that an increase in fluorescence at end point for molecular beacons with template over those with NTCs could be associated with a reduction in the melt temperature. Interaction between the products of LAMP amplification and the molecular beacons appear to give this marginal effect. However we would expect interaction with multiple copies of the target sequence and the specific molecular beacon to lead to a large increase in fluorescence due to unquenching, when compared to the quenched molecular beacon without template, this was not observed.

A LAMP molecular beacon in a PCR reaction failed to show increased fluorescence for the positive sample. The shorter probe sequence is likely to be a contributing factor to this. Conversely Liu et al. [10] showed that a typical PCR molecular beacon design failed to produce a fluorescent signal with LAMP amplification. This may be indicative of the annealing of the probe sequence to the amplicon interfering with LAMP amplification in real time. Furthermore the melt curve analysis from the PCR and LAMP amplifications with the LAMP molecular beacons showed an atypical profile for PCR molecular beacons in that at low temperature the presence of template did not increase proposed fluorescence from the molecular beacon.

It would be surprising that a molecular beacon could be used successfully in LAMP amplification. Perhaps therefore the increased fluorescence from the molecular beacons is the result of interactions with the LAMP concatemers. Whilst we cannot be sure that this increase in fluorescent signal with LAMP molecular beacons is a general effect applying in all combinations of LAMP amplicons and MBs, it seems clear that whilst they can, at appropriate concentration levels, provide an indication of amplification this does not result from sequence specificity but rather a more general feature of the LAMP amplicon.

## Conclusion

Molecular beacons for LAMP amplification targeting the stem region between loops are effective but non-specific reporters of LAMP amplification with both 35S promoter and NOS terminator primers. We found the optimum temperature for our assays to be 64^∘^C and achieved sensitivity comparable to the use of SYTO9. The mechanism by which the fluorescence from molecular beacons increases with LAMP amplification is unclear, although it appears that LAMP loop structures are important, but it is clear that there is no gain in specificity from the LAMP molecular beacons used in this study.

## Additional file

Additional file 1 Supplementary Information. (DOCX 299 kb)

## Data Availability

The datasets used and/or analysed during the current study are available from the corresponding author on reasonable request.

## Abbreviations

35Sp: 35S promoter
BART: Bioluminescent assay in real time
BLAST: Basic local alignment search tool
CRM: Certified reference material
Ct: Cycle threshold
DNA: Deoxyribonucleic acid
GC content: Guanine-cytosine content
HPLC: High-performance liquid chromatography
F3 and B3: Forward and backward displacement primers
FIP and BIP: Forward and backward inner primers
FRET: Fluorescence resonance energy transfer
GMO: Genetically modified organism
LAMP: Loop-mediated amplification
LoopF and LoopB: Forward and backward loop primers
LTR: Long terminal repeat
MB: Molecular beacons
NOSt: Nopaline synthase terminator
nt: Nucleotide
NTC: No template control
PCR: Polymerase chain reaction
Tm: Melt temperature
