# Supplementary material for: Lack of specificity associated with using molecular beacons in loop mediated amplification assays
Source: BMC Biotechnol. 2019 Aug 1;19:55. doi: 10.1186/s12896-019-0549-z (PMC6676609; doi:10.1186/s12896-019-0549-z)
Supplement: Supplementary file 1 — Supplementary Information. (DOCX 299 kb) [file 12896_2019_549_MOESM1_ESM.docx]

Supplementary Information

Lack of specificity associated with using molecular beacons in loop mediated amplification assays

**Patrick Hardinge^1,*^, James A. H. Murray^1^**

^1^Cardiff School of Biosciences, Biomedical Sciences Building, Museum Avenue, Cardiff CF10 3AX, UK

[*hardingep@cardiff.ac.uk](mailto:*hardingep@cardiff.ac.uk)


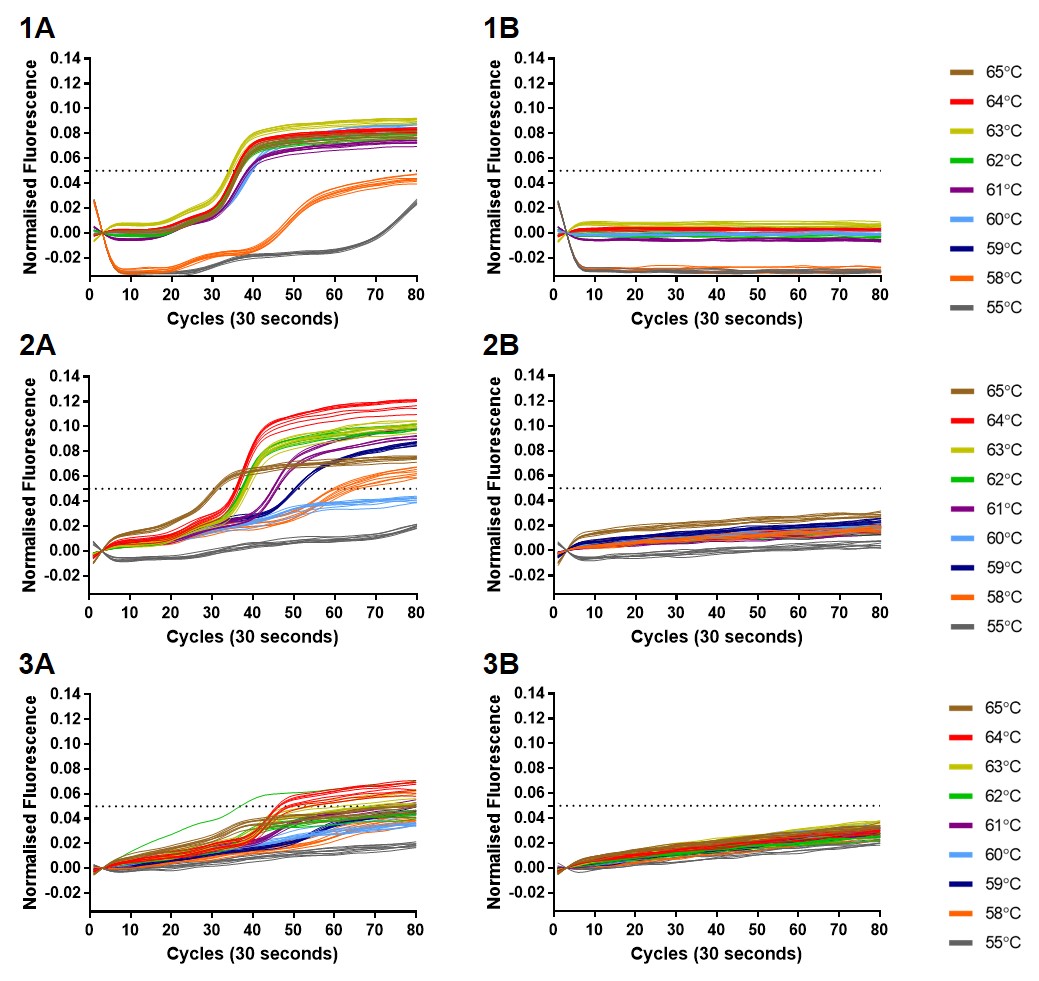


**Fig S1. Amplification curves of 35Sp STEM molecular beacons at different temperatures.**

LAMP assay temperature incrementally from 55°C to 65°C for positive template and NTC, (A) shows the increase in fluorescence from the excitation and unquenching of the FAM fluorophore attached to the 35Sp STEM molecular beacons at various temperatures and (B) shows the corresponding NTCs for each assay. 1A and 1B show the positive and negative samples at various temperatures for the 35Sp 4nt STEM MB, 2A and 2B show the results for 35Sp 6nt STEM MB and 3A and 3B for 35Sp 7nt STEM MB.


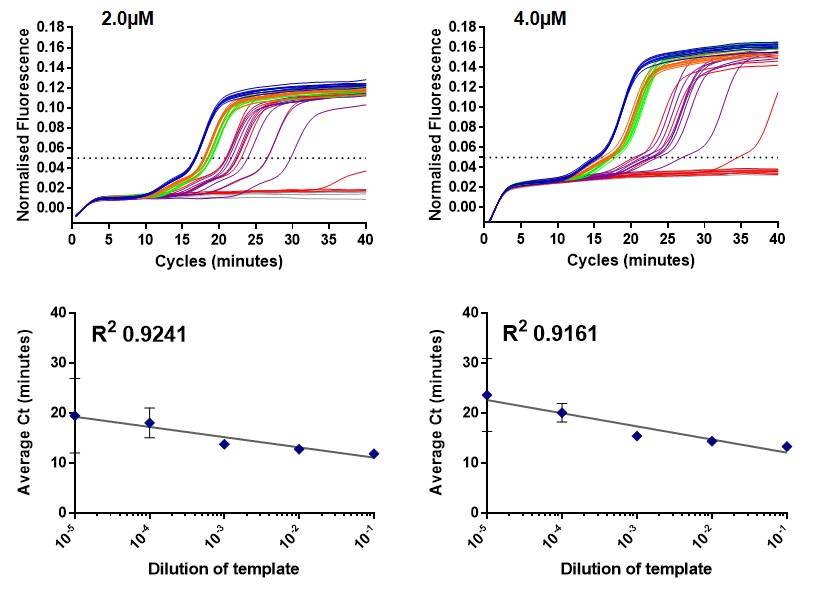


**Fig S2. Variation and separation between replicates in quantification.**

Fluorescent output from 35Sp 5nt STEM MB with 35Sp LAMP amplification of a serial dilution of plasmid template. Template dilutions: 10^-1^ dark blue, 10^-2^ orange, 10^-3^ light green, 10^-4^ purple, 10^-5^ red and NTC in grey. For each concentration of the molecular beacon the Ct value is derived from a set threshold of 0.05.
